# VEEV TC-83 Triggers Dysregulation of the Tryptophan–Kynurenine Pathway in the Central Nervous System That Correlates with Cognitive Impairment in Tg2576 Mice

**DOI:** 10.3390/pathogens13050397

**Published:** 2024-05-09

**Authors:** Chanida Fongsaran, Kelly T. Dineley, Slobodan Paessler, Irma E. Cisneros

**Affiliations:** 1 Department of Pathology, University of Texas Medical Branch, Galveston, TX 77555, USA; chfongsa@utmb.edu (C.F.); slpaessl@utmb.edu (S.P.); 2 Institute for Human Infections and Immunity, University of Texas Medical Branch, Galveston, TX 77555, USA; 3 Neuroinfectious Diseases, University of Texas Medical Branch, Galveston, TX 77555, USA; ktdinele@utmb.edu; 4 Mitchell Center for Neurodegenerative Diseases, Department of Neurology, University of Texas Medical Branch, Galveston, TX 77555, USA; 5 Center for Addiction Sciences and Therapeutics, University of Texas Medical Branch, Galveston, TX 77555, USA

**Keywords:** indoleamine 2,3-dioxygenase, neurodegeneration, quinolinic acid, Alzheimer’s disease, neuroinflammation, TC-83

## Abstract

Neurodegenerative diseases are chronic conditions affecting the central nervous system (CNS). Alzheimer’s disease (AD) is a neurodegenerative disorder characterized by the accumulation of amyloid beta in the limbic and cortical brain regions. AD is presumed to result from genetic abnormalities or environmental factors, including viral infections, which may have deleterious, long-term effects. In this study, we demonstrate that the Venezuelan equine encephalitis virus (VEEV) commonly induces neurodegeneration and long-term neurological or cognitive sequelae. Notably, the effects of VEEV infection can persistently influence gene expression in the mouse brain, suggesting a potential link between the observed neurodegenerative outcomes and long-term alterations in gene expression. Additionally, we show that alphavirus encephalitis exacerbates the neuropathological profile of AD through crosstalk between inflammatory and kynurenine pathways, generating a range of metabolites with potent effects. Using a mouse model for β-amyloidosis, Tg2576 mice, we found that cognitive deficits and brain pathology were more severe in Tg2576 mice infected with VEEV TC-83 compared to mock-infected controls. Thus, during immune activation, the kynurenine pathway plays a more active role in the VEEV TC-83-infected cells, leading to increases in the abundance of transcripts related to the kynurenine pathway of tryptophan metabolism. This pathway generates several metabolites with potent effects on neurotransmitter systems as well as on inflammation, as observed in VEEV TC-83-infected animals.

## 1. Introduction

Several arthropod-transmitted RNA viruses, which are zoonotic pathogens, are found in the genus *Alphavirus* in the family *Togaviridae*. Alphaviruses are characterized as positive-sense, single-stranded RNA viruses, commonly causing febrile illness followed by either encephalitic or arthralgia disease [1,2]. Venezuelan equine encephalitis virus (VEEV) is a mosquito-borne alphavirus that causes a highly virulent central nervous system (CNS) disease in horses and other equines with spill-over infections in humans [3,4]. In human cases of VEEV infection, approximately 4–14% of patients report neurological complications including seizures, photophobia, behavioral changes, neuromuscular weakness, and paralysis [5,6]. It is well documented that VEEV triggers neurological sequelae in survivors that can be characterized by cognitive, motor, and sensory deficits likely through direct neuronal and glial cell infection [7,8]. However, indirect mechanisms and biological components intertwined with the host CNS immune response to infection remain vague. 

Although somewhat controversial, increasing experimental and epidemiological evidence indicates that viral infections are an environmental risk factor for the development of neurodegeneration and neurological sequelae [9]. For example, neurotropic pathogens, such as human herpes virus (HHV), herpes simplex virus (HSV), hepatitis C virus (HCV), cytomegalovirus, varicella-zoster virus, and Epstein–Barr virus are associated with Alzheimer’s disease (AD) neuropathology [10,11,12,13]. Although previous studies do not indicate VEEV is related to neurodegeneration, VEEV neurological sequelae share progressive characteristics of neurodegenerative disorders including cognitive, motor, or sensory deficits [4,14,15]. 

Multiple preclinical and clinical studies indicate that VEEV leads to increased neuroinflammatory profiles and potential demyelination [7]. These immune responses are intertwined with other biological processes that can further influence the inflammation or metabolism of critical biological processes, thus impacting the brain [16]. One such pathway is the primary pathway of tryptophan (TRP) degradation. TRP is an essential amino acid precursor for the biosynthesis of the small endogenous molecules such as serotonin and numerous important CNS and immune system proteins. The kynurenine pathway (KP) is the major route of degradation of TRP [17]. During TRP degradation, the bioactive compound kynurenine (KYN) is produced and accounts for diverse biological functions, including regulation of the immune responsivity and involvement in immune-mediated disorders [18]. Several viral infections, including HIV [19], HCV [20], and HSV [21] increase activation of the KP, which interestingly is implicated in the neurological and cognitive impairments of patients with HIV, HCV, and HSV [21,22]. Moreover, inflammation induces the activation of indoleamine 2,3-dioxygenase (IDO) and diverts TRP metabolism from serotonin to KYN, reducing the availability of CNS-localized serotonin [23]. Triggering KYN levels results in the increased activation of several enzymes and generation of neuroprotective and neurotoxic metabolites including kynurenic acid (KYNA), 3-hydroxykynurenine (3-HK), and quinolinic acid (QUIN), ultimately leading to the generation of NAD+ [24,25,26]. The dysregulation of these enzymes and metabolites is implicated in neurodegenerative disorders and found to be elevated in the blood and CSF of AD patients [27]. This indicates KP activation during viral infections such as VEEV may drive the pathological interplay of inflammation and KYN dysregulation in the development or exacerbation of neurodegeneration [28,29,30].

**Scheme 1:**
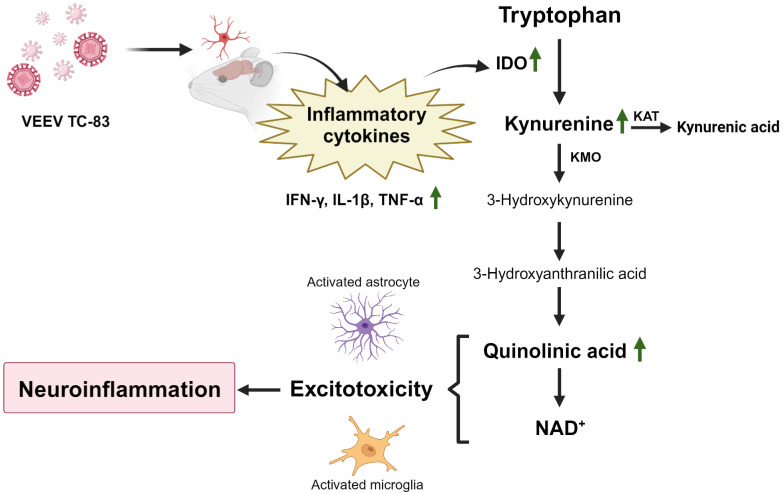
We propose that KP is implicated in inflammation-induced CNS pathology. Infection with VEEV TC-83 induces neuroinflammation and triggers the release of inflammatory cytokines such as IFN-γ, IL-1β, and TNF-α. These cytokines, in turn, activate the enzyme IDO, which converts TRP into KYN. The heightened activity of KP results in the synthesis of KYNA in astrocytes and the generation of QA in microglia. The activation of QA can subsequently stimulate NMDA receptors, increasing glutamate release and contributing to excitotoxicity, ultimately leading to neurodegeneration. Black arrows represent promoting function, and green arrows represent upregulation. Created with Biorender.com.

Here, we investigated whether CNS-localized KP dysregulation is triggered following infection with the alphavirus VEEV TC-83 (Scheme 1). The influence of host immune mediators, canonically activated by viral infections, increases IDO activity, and the metabolism of KYN is elevated. We further investigated whether the dysregulation of key KP metabolites correlates to inflammatory mediators and cognitive behavior which would further support the hypothesis that VEEV initiates neurological sequelae that are triggered by inflammation and KP dysregulation. Thus, adjusting KP activation could be a promising therapeutic approach for addressing cognitive impairment linked to elevated concentrations of KYNA in the brain. Additionally, understanding the contribution of KP production will be important in defining an appropriate intervention strategy in AD. 

## 2. Materials and Methods

### 2.1. Cells, Viruses, and Biosafety

Vero cells (American Tissue Culture Collection) were maintained in Dulbecco’s modified Eagle’s medium supplemented with 10% fetal bovine serum and 1% penicillin–streptomycin. TC-83 viral working stocks were produced in the Vero cells. All work with infectious viruses was performed at the University of Texas Medical Branch (UTMB) BSL-2 in accordance with institutional health and safety guidelines.

### 2.2. Animal Experiments 

The mice, both wild-type (WT) and Tg2576, were bred in the University of Texas Medical Branch (UTMB) Animal Care Facility by mating hemizygous Tg2576 male [Rd^−/−^] with (C57BL6/J × SJL/J)F1 female mice (The Jackson Laboratory). The offspring were genotyped via PCR of tail clippings taken within 21 days of age. UTMB complies with the U.S. Department of Agriculture Animal Welfare Act, the Guide for the Care and Use of Laboratory Animals, and Institutional Animal Care and Use Committee-approved protocols. The mice were housed in groups of five per cage with free access to food and water under a 12 h light/dark cycle, with controlled temperature and humidity. Male and female 3-months-old Tg2576 and WT mice were anesthetized using an isoflurane variable-bypass vaporizer prior to intranasal inoculation with 10^6^ PFU VEEV TC-83 diluted in phosphate-buffered saline (PBS) or PBS alone as the saline control. 

The Tg2576 model is one of the most well characterized and widely used mouse models of AD. It overexpresses a mutant form of APP (isoform 695) with the Swedish mutation (KM670/671NL), resulting in elevated levels of amyloid beta (Aβ) and ultimately amyloid plaques [31]. Tg2576 mice exhibit elevated Aβ from 2 months old onward, plaques after 6 months old, and cognitive deficits beginning at 5 months old [32,33,34]. Thus, at 3 months old, Tg2576 mice do not have detectable brain pathology or cognitive impairment and are considered at a prodromal stage of AD [35]. In certain assays, the sample size did not encompass all animals due to the unavailability of sufficient samples from each group. 

### 2.3. Active Avoidance

Active avoidance testing was performed at 6 months post-inoculation (mpi), in WT and Tg2576, uninfected and infected male and female mice, to evaluate their ability to learn using classic Pavlovian conditioning. The mice were handled throughout the 6 mpi, both during acute and chronic phases. Specifically, the mice were handled bi-weekly throughout the experiment timeline. The mice were removed individually from their home cages and placed into the Ugo Basile shuttle box, housed within a biosafety cabinet. The mice were stimulated with a sound (90–100 dB) and light (conditioned stimulus) for 10 s. Mice not moving to the other chamber during the conditioned stimulus received a shock (0.3 mA) for up to 4 s (unconditioned stimulus). The mice were allowed to escape the shock during these four seconds.

The mice performed 30 trials consecutively for 3 days. The number of avoidances, escapes, and the latency for avoidances and escapes were recorded. Mice that did not attempt to cross during the unconditioned stimulus 5 trials in a row were removed from the shuttle box and did not perform additional trials that day. 

### 2.4. Tissue Preparation

After the study (6 mpi), the mice were euthanized with carbon dioxide, and cervical dislocation was used to ensure death. All organs were rapidly extracted, frozen in liquid nitrogen, and stored at −80 °C until processing. Samples were homogenized using the TissueLyser II (Qiagen, Hilden, Germany) for 60 s at a speed of 3 m/s in 200–500 µL ice-cold homogenization/extraction buffer [20 mM HEPES (Cat# 25-060-CI; Corning, Corning, NY USA), 200 mM NaCl (Cat# 25915005; Corning), 1 mM EDTA (Cat# 46-034-CI; Corning), 1 mM DTT (Cat# 43815; Sigma-Aldrich, Darmstadt, Hesse, Germany), 10 µL/mL phosphatase inhibitor cocktail 2 (Cat# P5726; Sigma-Aldrich), 10 µL/mL phosphatase inhibitor cocktail 3 (Cat# P0044, Sigma-Aldrich), and RNase inhibitor (Cat# N8080119; Applied Biosystems, Foster City, CA, USA)]. Homogenized samples were aliquoted for RNA and protein isolation. The homogenates were centrifuged at 14,000× *g* for 5 min at 4 °C, and the supernatant was transferred to a new tube. The supernatant was collected and stored at −80 °C before protein or RNA extraction.

### 2.5. RNA Isolation and qRT-PCR

The total RNA was prepared using a standardized TRIzol reagent (Cat# 15596018; Invitrogen, Waltham, MA, USA) protocol. The RNA amount was assessed using spectrophotometer equipment (DeNovix DS-11+Spectrophotometer). First-strand cDNA was synthesized using the High-Capacity cDNA Reverse Transcription Kit (Cat# 4368814; Applied Biosystems) according to the manufacturer’s protocol. One microliter of cDNA templates was added to triplicate 20 µL reaction mixtures with TaqMan^®^ Fast Advanced Master Mix and primers (Cat# 4444557; Applied Biosystems). Quantitative PCR was then performed by StepOnePlus^TM^ Real-Time PCR System (Applied Biosystems) using TaqMan primer-probe sets and TaqMan reagents. The primer-probe sets used were mouse IDO1 (Mm00492589_m1), kynurenine (Mm00551012_m1), IFN-γ (Mm01168134_m1), and TNF-α (Mm00443258_m1), and rat IL-1β (Rr00580432_m1). The thermal profile was as follows: 95 °C for 2 min of initial denaturation followed by 40 cycles of 95 °C for 1 s to denature and 60 °C for 20 s to anneal and extend. The housekeeping gene, glyceraldehyde-3-phosphate dehydrogenase (GAPDH, Mm99999915_g1), was used as an internal control. The data were analyzed by calculating the 2^−ΔΔCt^ to determine the differences between the VEEV TC-83 and saline control groups in WT and Tg2576 mice.

### 2.6. Western Blot Analysis

The protein concentration of each sample was determined by a Precision Red Advance Protein Assay Reagent #2 (Cytoskeleton, Inc. Denver, CO, USA) following the manufacturer’s instructions. For each sample, an equal amount of protein (30 μg) was loaded onto 4–15% SDS polyacrylamide gels (Cat# 4568083; Bio-Rad, Hercules, CA, USA) and blotted onto a PVDF membrane (Cat# 1704274; Bio-Rad) using a Bio-Rad Mini-Blot transfer apparatus. The membranes were incubated with EveryBlot blocking buffer (Cat# 12010020; Bio-Rad). IDO (D8W5E) rabbit mAb (dilution 1:1000) (Cat# 51851S; Cell Signaling Technology, Danvers, MA, USA) was added to the membrane and incubated overnight at 4 °C. Membranes were then incubated with a 1:2000 secondary antibody Goat anti-rabbit IgG HRP (Cat# 7074P2; Cell signaling Technology) at room temperature for 1 h. Membranes were visualized using a chemiluminescence assay with Clarity Max Western ECL substrate (Cat# 1705062; Bio-Rad) and imaged on a Bio-Rad ChemiDoc^TM^ MP Imaging system (Bio-Rad). Band intensity was quantified using Image Lab software version 6.1.0 build 7 Standard Edition (Bio-Rad). The protein levels were normalized to the total protein for each sample.

### 2.7. Enzyme-Linked Immunosorbent Assay (ELISA)

ELISA for quinolinic acid (Cat# MBS2025601; MyBioSource, San Diego, CA, USA) was performed according to the manufacturer’s instructions (MyBioSource General Quinolinic Acid ELISA Kit). Briefly, equal concentrations of protein (30 µg) and standards were added into pre-coated 96-well plates and then incubated for 1 h at 37 °C. The plates were subsequently washed, and Detection Reagent B was added to each well for 30 min followed by the substrate and stop solution. The enzymatic reaction was quantified by measuring absorbance at 450 nm using a GloMax Discover Microplate reader (Promega, Madison, WI, USA). 

The kynurenine/tryptophan ratio was measured using the Kynurenine/Tryptophan ELISA pack (Cat# ISE-2227; Immusmol, Bordeaux, France) according to the manufacturer’s instructions, and their ratio was calculated and compared.

### 2.8. Enzyme Activity Assay

The rate of NAD^+^ was determined using a Sigma NAD^+^/NADH Assay Kit (Cat# MAK460; Sigma-Aldrich) according to the manufacturer’s protocol. Briefly, tissues were homogenized in NAD extraction buffer. The standard curve for NAD^+^ was used by serial dilution, and 50 µL standard and tissue lysate were used in a working solution. The concentration of NAD^+^ is determined by measuring the absorbance at λ_Ex_ = 530 nm/λ_Em_ = 585 nm. The total NAD^+^ concentration is calculated based on the standard curve.

IDO1 activity was measured with the IDO1 Activity Assay Kit (Cat# MAK356; Sigma-Aldrich), according to the manufacturer’s protocol. The tissues were prepared as previously described. The standard curve for IDO1 was used by serial dilution, and 50 µL standard and tissue lysate were used in a working solution. The concentration of IDO1 is determined by measuring the absorbance at λ_Ex_ = 402 nm/λ_Em_ = 488 nm. The IDO1 activity is calculated based on the standard curve.

### 2.9. Statistical Analysis

Two-sided power analysis indicates a minimum sample size of 10 animals per group. Based on the means and variances of the majority of the behavior data we collected, and to account for infection-related variability, we included a sample size between 10–16 animals per group. Statistics were performed using GraphPad Prism (version 10.0.2). For analyzing the differences in multiple groups, comparisons were performed using a one-way analysis of variance (ANOVA) followed by Tukey post-hoc analysis. All data are presented as mean ± SEM. A significance level of *p* < 0.05 at 95% confidence intervals was considered statistically significant for all the experiments reported in this study.

## 3. Results

### 3.1. Increased IDO Levels in CNS Are Associated with VEEV TC-83 Infection

**Figure 1:**
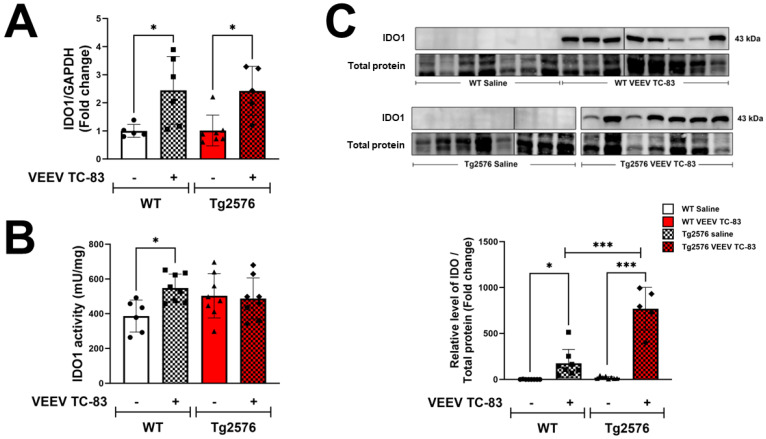
Increased IDO levels in CNS are associated with VEEV TC-83 infection. (**A**) Total brain tissue homogenates collected at 6 mpi were assayed from changes of expression in transcripts encoding IDO. (**B**) IDO1 activity was measured in VEEV TC-83-inoculated WT and Tg2576 mice and normalized to total protein concentrations. (**C**) IDO protein levels were evaluated by Western blot using a monoclonal antibody specific for IDO and normalized to total protein levels. Densitometry analysis represents the ratio of the relative abundance of IDO1 to total protein for WT saline (*n* = 5–7), WT VEEV TC-83 (*n* = 6–8), Tg2576 saline (*n* = 7–8), and Tg2576 VEEV TC-83 (*n* = 5–8) mice. The results were then plotted on a graph. Images obtained using stain-free gel technology (Bio-Rad), which allows for total protein visualization and quantification, are shown as a loading control. Each bar represents the mean ± SEM of the results from mice. * *p* < 0.05, and *** *p* < 0.001.

Increased IDO expression is responsible for the catabolism of TRP in the KYN degradation pathway [36] and is canonically upregulated by inflammatory mechanisms [37,38]. Thus, to determine the presence of IDO, the first and rate-limiting enzyme in the KP, we quantified expression at the RNA and protein levels. We measured increased IDO transcripts in total brain homogenates of VEEV TC-83-inoculated WT and Tg2576 mice at 6 months post-inoculation (mpi). IDO transcript levels were approximately 2.4-fold higher in VEEV TC-83-inoculated WT mice compared to WT mice inoculated with saline (*n* = 11; * *p* < 0.05, Figure 1A). Inoculation with VEEV TC-83 also significantly increased IDO transcripts by approximately 2.4-fold in Tg2576 mice (*n* = 12; * *p* < 0.05, Figure 1A). We did not measure any significant differences between the WT or Tg2576 inoculated mice. To investigate IDO enzyme activity in the brain, we measured IDO functional activity in total brain homogenates by quantifying the enzymatic activity of IDO using L-tryptophan as a substrate in both the saline control and VEEV TC-83-inoculated WT and Tg2576 mice. There was significant upregulation of IDO1 activity in VEEV TC-83-inoculated WT mice, increasing from 386.28 pmole to 548 pmole of IDO activity (*n* = 14, * *p* < 0.05, Figure 1B). There was no significant difference in IDO1 activity in Tg2576 mice. IDO protein levels were also significantly increased in WT mice inoculated with VEEV TC-83 (*n* = 16; * *p* < 0.05, Figure 1C). Interestingly, Tg2576 mice inoculated with VEEV TC-83 had an approximately 40-fold increase in IDO levels compared to saline-inoculated Tg2576 mice (*n* = 13; *** *p* < 0.001, Figure 1C) and significantly higher IDO levels compared to VEEV TC-83-inoculated WT mice (*n* = 13; *** *p* < 0.001, Figure 1C).

### 3.2. IDO Levels Correlate to Inflammatory Mediators and Active Avoidance Behavior

**Figure 2:**
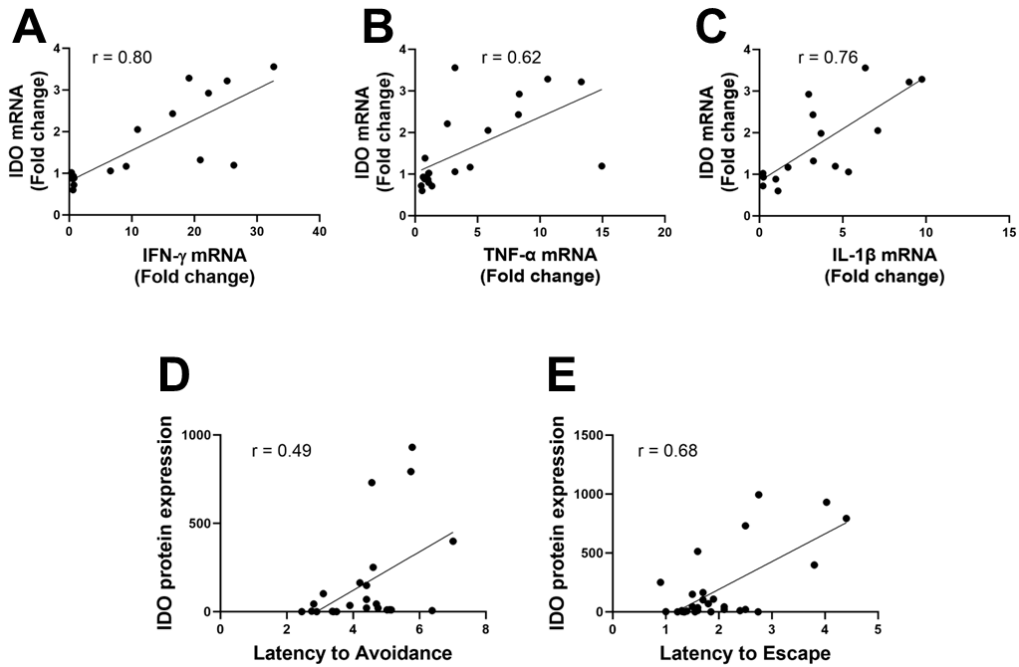
IDO levels correlate to inflammatory mediators and active avoidance behavior. The distribution of transformed, combined data for (**A**) IDO mRNA vs. IFN-γ mRNA (R^2^ = 0.6340; *** *p* < 0.001; *n* = 16); (**B**) IDO mRNA vs. TNF-α mRNA (R^2^ = 0.3845; ** *p* < 0.01; *n* = 21); and (**C**) IDO mRNA vs. IL-1β mRNA (R^2^ = 0.5814; *** *p* < 0.01; *n* = 16) was analyzed by linear regression analysis and evaluated for correlational relationships. The distribution of protein levels for IDO vs. the latency to avoid ((**D**), R^2^ = 0.2367; * *p* < 0.05; *n* = 25) or escape ((**E**), R^2^ = 0.4606; *** *p* < 0.001; *n* = 29) shock was analyzed by linear regression analysis and evaluated for correlational relationships. All correlations were analyzed using Pearson’s correlation.

Further analysis identified correlations in the CNS between the expression of IDO mRNA and inflammatory cytokine gene expression following VEEV TC-83 inoculation in WT and Tg2576 mice. We previously demonstrated a robust immune phenotype in the CNS and altered learning and memory in a chronic model of VEEV TC-83 infection [39]. In these experiments, the results show a significant correlation of IDO mRNA and IFN-γ mRNA (R^2^ = 0.6340; *** *p* < 0.001; *n* = 16, Figure 2A); IDO mRNA and TNF-α mRNA (R^2^ = 0.3845; ** *p* < 0.01; *n* = 21, Figure 2B), and IDO mRNA and IL-1β mRNA (R^2^ = 0.5814; *** *p* < 0.01; *n* = 16, Figure 2C). The IDO protein expression levels also significantly correlated with latency to avoid (R^2^ = 0.2367; * *p* < 0.05; *n* = 25, Figure 2D) or escape the shock (R^2^ = 0.4606; *** *p* < 0.001; *n* = 29, Figure 2E).

### 3.3. Tryptophan Catabolism and KP Activation Are Upregulated during Infection

**Figure 3:**
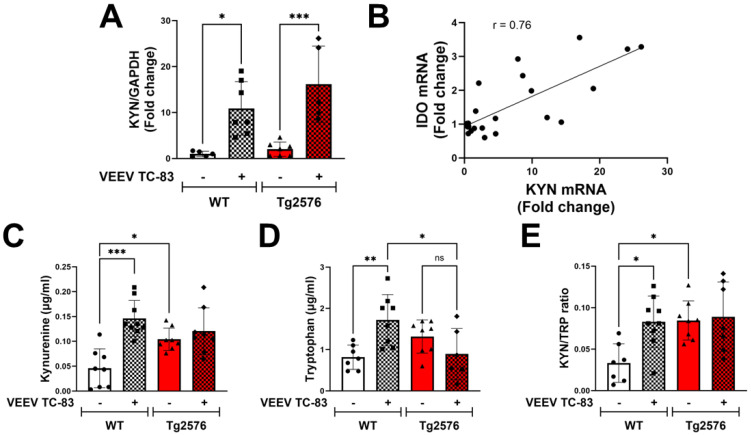
TRP catabolism and KP activation are regulated by VEEV TC-83 exposure. (**A**) Total brain homogenates collected at 6 mpi were assayed for KYN expression for WT saline (*n* = 5–8), WT VEEV TC-83 (*n* = 7–9), Tg2576 saline (*n* = 7–8), and Tg2576 VEEV TC-83 (*n* = 5–8) mice. (**B**) The distribution of transformed, combined data for IDO mRNA vs. KYN mRNA (R^2^ = 0.5717; *** *p* < 0.001; *n* = 22) were analyzed by linear regression analysis and evaluated for correlational relationships. Total protein levels of (**C**) KYN and (**D**) TRP were quantified in total brain homogenates following 6 mpi from saline- or VEEV TC-83-inoculated WT and Tg2576 mice. (**E**) Ratios of total protein levels of KYN and TRP were quantified. All correlations were analyzed using Pearson’s correlation. Each bar represents the mean ± SEM. * *p* < 0.05, ** *p* < 0.01, and *** *p* < 0.001.

KYN is regulated by the levels and functional activity of IDO; thus, we quantified KYN mRNA expression in total brain homogenates. We found significantly increased KYN transcripts in the total brain lysates of in both VEEV TC-83-inoculated WT and Tg2576 mice. Specifically, KYN transcript levels were approximately 11-fold higher in VEEV TC-83-inoculated WT mice compared to saline-inoculated mice (*n* = 12; * *p* < 0.05, Figure 3A). Inoculation with VEEV TC-83 also significantly increased KYN levels approximately eight-fold in Tg2576 mice (*n* = 12; *** *p* < 0.001, Figure 3A). Our findings indicate a positive correlation between IDO and KYN expressions in both the saline control and in VEEV TC-83-inoculated mice (*n* = 22; *** *p* < 0.001, R^2^ = 0.5717, Figure 3B).

We compared the concentrations of TRP and KYN between saline-inoculated and VEEV TC-83-inoculated WT and Tg2576 groups. The mean KYN concentration in WT mice ranged from 0.046 µg/mL to 0.146 µg/mL, approximately three-fold, showing a significant increase initiated by VEEV TC-83 (*n* = 17; *** *p* < 0.001, Figure 3C). No significant differences were found in the levels of KYN in the Tg2576 mice, whether saline- or VEEV TC-83-inoculated. Interestingly, Tg2576 mice inoculated with saline had an approximate 2.26-fold increase in KYN levels compared to WT saline-inoculated mice, ranging from 0.046 µg/mL to 0.104 µg/mL (*n* = 16; * *p* < 0.05, Figure 3C). TRP concentration increased by two-fold, from a mean of 0.814 µg/mL in saline-inoculated WT mice to 1.718 µg/mL in VEEV TC-83-inoculated WT mice (*n* = 15; ** *p* < 0.01, Figure 3D). However, we did not observe significant differences between saline-inoculated and VEEV TC-83-inoculated Tg2576 mice. In contrast, the TRP concentration in VEEV TC-83-inoculated Tg2576 mice was notably lower, with a decrease of approximately 1.9-fold from 1.718 µg/mL to 0.891 µg/mL, compared to VEEV TC-83-inoculated WT mice (*n* = 14; * *p* < 0.05, Figure 3D). To further investigate differences in IDO and immune activation triggered by VEEV TC-83 in both WT and Tg2576 mice, the KYN/TRP ratio was calculated. The KYN/TRP ratio was significantly increased in VEEV TC-83-inoculated WT mice (Figure 3E). Specifically, mean values of 0.033 µg/mL in saline-inoculated mice and 0.083 µg/mL in VEEV TC-83-inoculated WT mice were measured (*n* = 16; * *p* < 0.05, Figure 3E). However, Tg2576 mice inoculated with saline showed a significant increase in KYN/TRP ratios compared to WT saline-inoculated mice (*n* = 15, * *p* < 0.05, Figure 3E). There was no statistically significant difference in the KYN/TRP ratio observed in VEEV TC-83-inoculated Tg2576 mice compared to saline-inoculated Tg2576 mice.

### 3.4. VEEV TC-83 Impacts QUIN and NAD^+^ Levels in Tg2576 Mice Leading to Behavioral Changes

**Figure 4:**
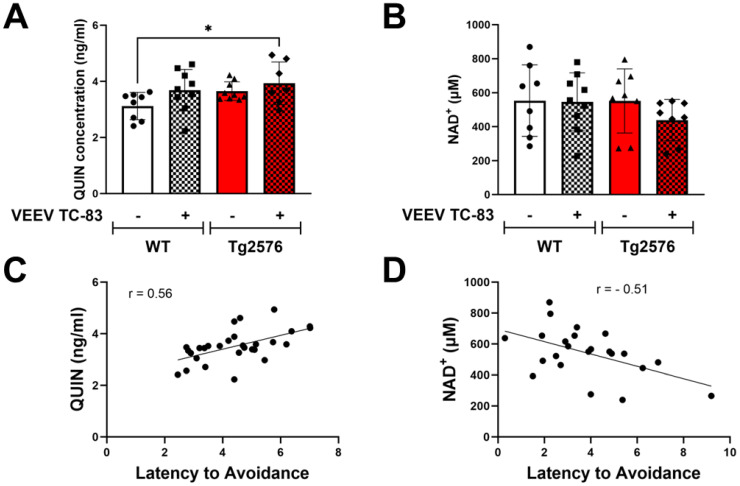
VEEV TC-83 impacts QUIN and NAD^+^ levels in Tg2576 mice, impacting behavior. (**A**) Total QUIN and (**B**) NAD^+^ levels were quantified in total brain homogenates from WT and Tg2576 mice 6 months post-inoculation. The sample groups consisted of WT saline (*n* = 8), WT VEEV TC-83 (*n* = 9), Tg2576 saline (*n* = 8–9), and Tg2576 VEEV TC-83 (*n* = 7–8) mice. The distribution of transformed, combined data for (**C**) QUIN vs. the latency to avoid shock (R^2^ = 0.3140; ** *p* < 0.01; *n* = 29) and (**D**) NAD+ vs. the latency to avoid shock (R^2^ = 0.2556; * *p* < 0.05; *n* = 23) were analyzed by linear regression analysis and evaluated for correlational relationships. All correlations were analyzed using Pearson’s correlation. Each bar represents the mean ± SEM of the results from mice. * *p* < 0.05.

KYNA and QUIN represent the end products of the two primary branches of the KP, each released at varying concentrations. The conversion of KYN into various downstream metabolites, including 3-HK, 3-HAA, and QUIN, is associated with neurotoxic properties downstream of KMO activity. We also measured the amount of QUIN, which serves as a marker for the stage of excitotoxicity in KP metabolites. QUIN concentrations were significantly increased in Tg2576 VEEV TC-83-inoculated mice, ranging from 3.12 ng/mL to 3.93 ng/mL, which was 1.3-fold higher than in saline-inoculated WT mice (*n* = 15; * *p* < 0.05, Figure 4A). This indicates the presence of neurotoxicity in the brains of Tg2576 mice. As QUIN can serve as a precursor for NAD^+^, we assessed the functional consequences of QUIN on NAD^+^. While there was no significant difference in NAD^+^ concentrations in the brain between saline-inoculated and VEEV TC-83-inoculated mice, we observed a slight decrease, which was not statistically significant, in VEEV TC-83-inoculated Tg2576 mice (Figure 4B). These findings suggest that QUIN accumulation in VEEV TC-83-inoculated mice leads to reduced NAD^+^ activity. Correlation analysis was used to determine the relationship between QUIN, NAD^+^ activity, and behavior. The positive significant correlation between QUIN and latency to avoid shock suggests that increased QUIN production is correlated with increases in latency to avoid shock (R^2^ = 0.3140; ** *p* < 0.01; *n* = 29, Figure 4C). Further analysis identified an inverse correlation between NAD^+^ and latency to avoid shock, suggesting that increased NAD^+^ production could counteract latency for active avoidance (R^2^ = 0.2556; * *p* < 0.05; *n* = 23, Figure 4D).

## 4. Discussion

In this study, we tested the hypothesis that CNS KP dysregulation, following VEEV TC-83 exposure in WT and Tg2576 mice, is correlated to altered inflammatory profiles and behavior outcomes. We previously demonstrated a robust immune phenotype in the CNS and altered learning and memory outcomes that align with the neuropathology of AD, in a chronic model of VEEV TC-83 infection [39]. By utilizing a preclinical AD rodent model, the Tg2576 mouse, versus its wild-type control, we anticipated that inoculation with an alphavirus would support the infectious etiology of the AD hypothesis, which proposes that pathogens are the root cause of AD and is supported by emerging evidence that infection with neurotropic viruses is related to cognitive decline in AD [40]. In parallel, viral infection prior to AD diagnosis may accelerate the manifestation of AD, while viral infection during AD, as tested in this study, may accelerate AD progression. This is likely multifactorial but triggered by the host immune response to viruses. Importantly, the Tg2576 mouse model exhibits progressive cognitive decline and AD-like pathology, yet neither profound inflammation nor neurodegeneration is evident, suggesting that additional sequelae, like those initiated by neurotropic viruses, are necessary to manifest full disease profiles. Furthermore, as the KP is triggered by immunological events, here we first explore the transcriptional activity of genes responsible for encoding the critical enzymes involved in the KP, given that the breakdown of TRP along the KP mediates the generation of the neuroactive metabolites KYNA and QUIN, which are linked to a variety of neurodegenerative diseases [41]. Moreover, in line with other infectious disease studies, other neurotropic viruses, including chikungunya virus (CHIKV), activate the KP, through exerting antiviral effects and exacerbating inflammatory responses [42]. Therefore, an explanation for the neuropathologic alterations seen in the brains of infected mice in both WT and Tg2576 mice with VEEV TC-83 could be found in the differential regulation of the KP. These findings provide biological insights into the potential role of KP dysregulation following infection with neurotropic viruses and CNS immune activation in neurodegenerative diseases. 

We observed an increase in the neuroinflammatory marker, IDO, following VEEV TC-83 infection, and found IDO to be significantly heightened in Tg2576 mice infected with VEEV TC-83, suggesting that in AD, there is heightened sensitivity to IDO activity, which may be triggered via heightened neuroinflammatory pathways. Notably, IDO increases were significantly enhanced in parallel to increased levels of IFN-γ, IL-1β, and TNF-α, in both WT and Tg2576 mice, further suggesting that IDO activity likely follows innate immune responses to VEEV TC-83 infection and serves as the initiation step of TRP metabolism in the CNS [43]. This aligns with the notion that inflammatory conditions, despite their generation, stimulate TRP degradation and are related to increased levels of KP metabolites in the brain [23], including the neuroprotective KYNA and neurotoxic QUIN [44]. This is further supported by clinical studies identifying heightened IDO activity, increased KYN/TRP ratios, and increase concentrations of TNF-α, IL-6, IFN-γ, CCL2, and CXCL10 in CHIKV patients [42]. Interestingly, we found a modest positive correlation between IDO levels and abnormal behavioral phenotypes following VEEV TC-83 infection. Specifically, we found that Tg2576 VEEV TC-83-inoculated mice had an increased latency to avoid or escape shock they also had increasing levels of IDO activity. These results suggest that IDO may be associated with the development of neurological damage, playing important roles in behavioral impairment affecting behavioral performance. While we do not demonstrate that infection, inflammation, and IDO activation are causative of behavioral abnormalities and neurological sequalae following VEEV infection, the strong correlation provides evidence for future studies. 

As an expected outcome of increased IDO activity is increased KYN, we found that despite the genetic background of the mice, VEEV TC-83 initiated KYN transcription. Given that IDO activity and levels align with KYN levels, we expected to quantify a significant increase of KYN levels in infected Tg2576 mice compared to infected WT mice, but only observed a trending increase. This expectation is teased out when we measured increased KYN protein levels in Tg2576 mice compared to WT mice in the absence of VEEV TC-83. This is likely due to the heightened baseline level of inflammatory cytokines in Tg2576 mice, given that these mice are predisposed to acquire amyloid beta tangles and AD neuropathology [45]. We anticipated that TRP levels would be reduced in infected mice, since TRP degradation is diverted to KYN following IDO activity [46]; however, we found increased TRP levels in infected WT mice that was absent in infected Tg2576 mice. Alternatively, in infected Tg2576 mice the decrease in TRP concentrations was more pronounced than increases in KYN, which may be attributed to the rapid metabolism of KYN once it is produced [47]. The KYN/TRP ratio, particularly when comparing the concentration of IDO’s first product to the substrate, is a reliable indicator for tracking TRP degradation, which further implies enhanced IDO activity [48]. We measured increased KYN/TRP ratios in WT mice infected with VEEV TC-83 and in Tg2576 mice when compared to uninfected WT mice. The absence of KYN, TRP, or KYN/TRP ratios in Tg2576 mice infected with VEEV TC-83 may be a result of saturated KP signaling stemming from heightened neuroinflammatory pathways in Tg2576 mice. Previous work demonstrates that the response of microglia around amyloid plaques in the Tg2576 mouse trigger increased IDO activity because of inflammation [49]. The observed relationship between VEEV TC-83 infection and the progression of AD may contribute to the development of AD, leading to elevated levels of Aβ and the formation of amyloid plaques [31]. This indicates a relationship between all outcomes and the levels of KP metabolites in the CNS, showing a significant enhancement of downstream metabolites. 

Our studies are specifically focused on the neurotoxic outcomes related to VEEV TC-83 infection; therefore, we quantified the end products of the KP, which include QUIN and NAD^+^ [50]. QUIN is a neurotoxin that can induce apoptosis in neurons, oligodendrocytes, and astrocytes, which is characteristic of neurodegenerative disorders [51,52]. Neurotoxicity in neurons is triggered by the overstimulation of NMDA receptors, leading to disruptions in intracellular Ca^2+^ homeostasis and free radical formation [53,54]. This mechanism is associated with previous studies showing that quinolinic acid (QA), a QUIN precursor, contributes to neuroinflammation and is implicated in various neurodegenerative diseases [55,56,57]. We expected to find that QUIN levels would be closely related to levels of IDO, KYN, and/or KYN/TRP ratios, but we found no significant differences in QUIN levels following infection in either WT or Tg2576 mice. We only found significantly increased levels of QUIN to be present in the infected Tg2576 mice when compared to uninfected WT mice. This suggests that in the Tg2576 mouse model of AD, characterized by β-amyloid accumulation, QUIN may contribute to increased KYN signaling [18,58]. Furthermore, QUIN is transiently produced and metabolized into NAD^+^ in response to inflammation and infection [59,60]. Several studies indicate elevated QUIN levels in leukocytes, during inflammatory responses, contribute to increased NAD^+^ [61,62]. However, we found no significant differences in NAD^+^ activity between WT and Tg2576 mice in both the saline- and VEEV TC-83-inoculated groups. These findings suggest that abnormal KP function in the brain may be causally involved during VEEV infection. Although levels of QUIN and NAD^+^ were subtle, or unchanged, we found that the trends in QUIN and NAD^+^ changes correlated to avoidance behaviors in WT and Tg2576 mice. Particularly, NAD^+^ exhibited a modest negative correlation, while QUIN showed a slight positive correlation. Thus, taken together, we found that both QUIN and NAD^+^ are possibly involved in the acquisition and contribute to behavioral changes influencing the learning of active avoidance responses, resulting in delayed response latencies. This reflects a cautious approach and suggests a notable decline in spatial ability and memory dysfunction. 

## 5. Conclusions

Our studies implicate a mechanism of KP involvement in neuropathogenesis, directing KP metabolism toward the neurotoxic metabolite QUIN and limiting neuroprotection. These findings indicate that cytokines are upregulated in response to VEEV TC-83 and mediate the induction of IDO expression. The observed changes in KP metabolites, such as QUIN and NAD^+^, suggest a differential activation of the KP that may play a role in the progression and severity of virus infection. Hence, additional research focusing on the enzymes responsible for catalyzing the production of various KP metabolites, as well as exploring potential KP enzyme inhibitors, which emerge as a promising avenue for therapeutic interventions in neurodegenerative diseases, is warranted.

## Data Availability

Data will be made available on request.
